# Supplementary material for: Metabolic Responses of the Microalga Neochloris oleoabundans to Extracellular Self- and Nonself-DNA
Source: Int J Mol Sci. 2023 Sep 16;24(18):14172. doi: 10.3390/ijms241814172 (PMC10531809; doi:10.3390/ijms241814172)

**Supplementary Materials:**

Table S1. Parameters of the phytohormone's validation.

| Component | RT (min) | Scan | Transition SRM | CE (eV) | Slope (b) | Interception (a) | $R^2$ | Sx/y | LOD (pM) | LOQ (pM) |
|---|---|---|---|---|---|---|---|---|---|---|
| ***tZ/cZ*** | 7.55/8.90 | + | 220.11 → 202.03 | 35 | 5880.7 | 2409.7 | 0.9998 | 17108.27 | 8.73 | 29.09 |
| **SA** | 12.39 | - | 137.02 → 93.03 | 35 | 81.0 | 6011.9 | 0.9998 | 364.52 | 13.50 | 44.99 |
| **GA3** | 14.39 | - | 345.13 → 239.22 | 35 | 8640.1 | 14753.4 | 0.9999 | 3612.11 | 1.25 | 4.18 |
| **IAA** | 14.41 | + | 176.07 → 130.06 | 35 | 866.2 | -6126.5 | 0.9999 | 2271.45 | 7.87 | 26.22 |
| **IsoA** | 15.26 | + | 204.12 → 136.06 | 35 | 3233.8 | 5317.4 | 0.9999 | 4738.97 | 4.40 | 14.65 |
| **BAP** | 15.41 | + | 226.10 → 91.05 | 35 | 236.4 | 73687.2 | 0.9999 | 1989.26 | 2.24 | 7.47 |
| **ABA** | 17.27 | - | 263.12 → 219.14 | 35 | 236.4 | -510.6 | 0.9941 | 3282.18 | 41.64 | 138.79 |
| **MeJA** | 20.33 | + | 225.14 → 207.05 | 35 | 18185.3 | 503680.5 | 0.9998 | 53446.24 | 8.82 | 29.39 |

Retention time in min, (RT); Selected reaction monitoring, (SRM); Electrical current, (CE) in electrovolts (eV); Slope and intercept of the linear regression, (**b** and **a)** respectively; Standard deviation of the response, (Sx/y); Limit of detection (LOD); Limit of quantification (LOQ)

Figure S1

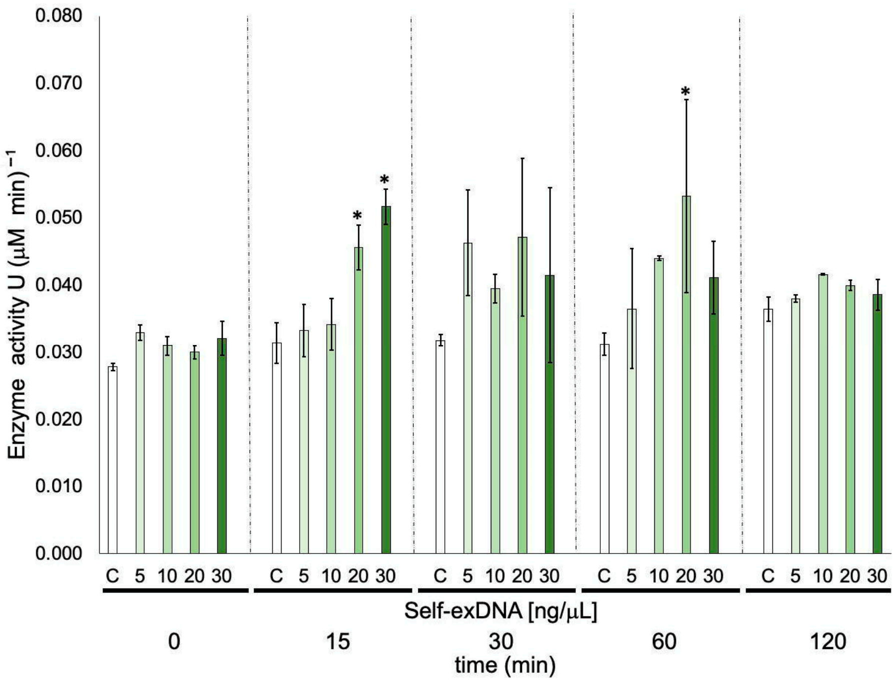


**Figure S1.** Dose-response curve of the peroxidase enzyme activity of *Neochloris oleoabundans*, after application of self-exDNA at different concentrations and times. The unit of enzyme activity (U) was defined as the catalytic activity responsible for the transformation of one μmol substrate per minute under optimal enzyme conditions. (c), control without self-exDNA. Data represent mean ± standard deviation (SD, n = 3). Asterisks indicate statistically significant differences (* = $p < 0.05$; ** = $p < 0.01$; *** = $p < 0.001$).

Figure S2

Standard curves of phytohormones

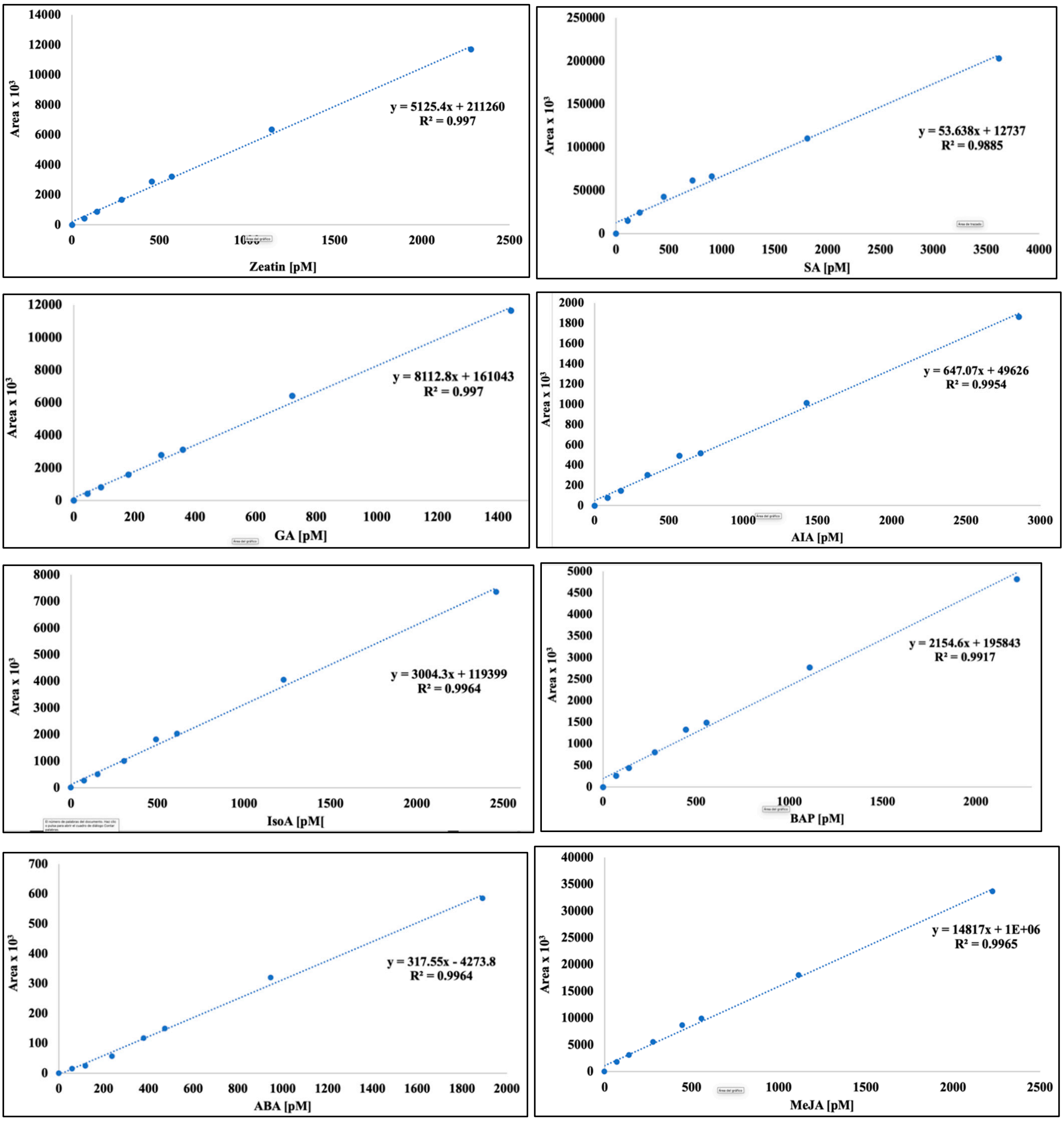

Supplement: Supplementary file 1 [file ijms-24-14172-s001.zip › ijms-2529882-supplementary.pdf]
